# A review on ocular findings in mouse lemurs: potential links to age and genetic background

**DOI:** 10.5194/pb-4-215-2017

**Published:** 2017-10-27

**Authors:** Marko Dubicanac, Ute Radespiel, Elke Zimmermann

**Affiliations:** Institute of Zoology, University of Veterinary Medicine Hannover, Buenteweg 17, 30559 Hannover, Germany

## Abstract

Mouse lemurs, the world's smallest primates, inhabit forests in Madagascar. They are nocturnal, arboreal and dependent on vision for their everyday lives. In the last decades, the grey mouse lemur became increasingly important for biomedical research, in particular aging research. Experiments which require the combination of visual fitness and old age consequently depend on a solid knowledge of ocular pathologies. Although ocular diseases in mouse lemurs have been described as being common, they have not received much attention so far. Yet it is important to know when and why ocular diseases in captive mouse lemurs may occur. This review aims to provide a comprehensive overview of known ocular findings in mouse lemurs. It summarizes the frequency of ocular findings in captive mouse lemur colonies and points to their likely causes and treatment options based on the evidence available from other animals and humans. In addition, it shall be discussed whether age or genetic background may affect their development. This review may be used as a reference for future studies which require an assessment of visual performance in mouse lemurs and help to evaluate observed clinical signs and ocular diseases. Furthermore, the high incidence of specific diseases may provide new perspectives and set the groundwork for a new animal model for ocular research.

## 1 Introduction

The mouse lemur is an endemic primate from Madagascar, which belongs to the smallest living primates worldwide (Mittermeier et al., 2010). The small body size, young age at first reproduction (ca. 8 months) and high reproductive potential (up to 2–3 litters of twins per year) makes these non-human primates (NHPs) much more attractive and cost-efficient for maintenance and breeding than larger primates. Their maximum life expectancy of about 8 years in the wild (Zimmermann et al., 2016) and up to 18.5 years in captivity (Weigl and Jones, 2005) is much shorter than for other non-human primates (Weigl and Jones, 2005; Zimmermann and Radespiel, 2015). Consequently, mouse lemurs represent an important animal model for aging (Perret, 1997; Cayetanot et al., 2005; Gomez et al., 2012; Languille et al., 2012; Zimmermann and Radespiel, 2014; Zimmermann et al., 2016) and biomedical research, e.g. on Alzheimer's disease (Austad and Fischer, 2011; Verdier et al., 2015). Nevertheless, based on their high cryptic species diversity, their uneven geographic distribution and their socioecological plasticity, mouse lemurs are also of major interest for evolutionary research (Zimmermann and Radespiel, 2014). Additionally, the genome of mouse lemurs has recently been sequenced by the Broad Institute (GenBank accession number ABDC00000000), underlining the importance of this animal model for research. Mouse lemurs are nocturnal and therefore possess relatively large eyes (9.4±0.5 mm in diameter) compared to their total skull size (Kirk, 2004; Ross and Kirk, 2007). The relatively high life expectancy in captivity (compared to animals living in the wild) makes these animals vulnerable to a variety of eye diseases and injuries. Some of these pathologies have already been described (Beltran et al., 2007), but it is unknown whether the described ocular findings are limited to two colonies originating from the same founder individuals or are more widespread across aging colonies of this species. With regard to the increasing relevance of mouse lemurs for evolutionary and biomedical research, an updated overview of known ocular disorders and their potential impact on vision as well as their respective medical treatment is necessary.

The purpose of this review is therefore (1) to characterize and summarize ocular findings described in large mouse lemur colonies, (2) to examine whether age may have an influence on the development of specific ocular findings, and (3) to identify a potential genetic background of the described ocular findings.

## 2 Methods

### 2.1 Literature review

Three international literature databases (Web of Science, Google Scholar and PubMed) were systematically screened for the current state of knowledge on ocular findings in mouse lemurs. Keywords which have been used for searching in different combinations were *mouse lemur, lemur, primate, Cheirogaleidae, pathology, disorder, impairment, disease, ocular, ocular disease, eye* and *eye disease*. This search resulted in one publication, describing a study on 218 grey mouse lemurs (*Microcebus murinus*) in two different colonies belonging to the Muséum National d'Histoire Naturelle in Paris and in Brunoy (Beltran et al., 2007) and two published studies from our group (Dubicanac et al., 2016, 2017).

### 2.2 Empirical data

To enlarge the present knowledge on the diversity of ocular findings in the grey mouse lemur (*Microcebus murinus*), we add information on all diagnosed cases which have been found during ophthalmological investigations at the Institute of Zoology, University of Veterinary Medicine Hannover, Germany between the years 2012 and 2016 (partially published in Dubicanac et al., 2016, 2017). All in all, data included repeated inspections of 100 animals (49 males, 51 females) with ages ranging from 8 months to 13.6 years.

The ophthalmological study was licensed by the respective authority (Hannover licence number, 33.9-42502-05-11A200, LAVES to Elke Zimmermann) and complies with animal care regulations and the applicable national law, and adheres to the legal requirements.

## 3 Results

### 3.1 Overview of ocular findings

A summary of all findings is shown in Table 1. The most frequent ocular findings by far were cataracts and nuclear sclerosis, followed by corneal degeneration, synechiae and pupil seclusion. All other ocular findings were documented only 1–3 times and were thus rare.

**Table 1:** Summary of all published ocular findings from mouse lemur colonies.

Ocular finding	Beltran et al. (2007) (n=218 animals screened)– number of affected individuals	This study (n=100 animals screened)– number of affected individuals
Cataracts	66	34
Nuclear sclerosis	–	45
Pupil seclusion	6	5
Synechiae	9	1
Hyphema	1	1
Intraocular hypertension	–	1
Exophthalmos	–	1
Buphthalmia	1	–
Phtisis bulbi	1	1
Ectopia lentis	1	1
Corectopia	1	–
Thrichiasis	–	1
Corneal dystrophy	3	–
Corneal degeneration	9	–
Chorioretinitis (scars)	1	–

### 3.2 Ocular findings with definition, diagnosis, potential treatment, incidence and potential link to age and genetic background

#### 3.2.1 Cataracts

#### Definition

Cataracts is a disease of the lens. It is a visual cloudiness in the lens (Gelatt et al., 2012; Maggs et al., 2012), which is caused by changes in cellular proteins and arrangement, which lead to light-scattering structures. Besides age, UV light and genetic background, as well as oxidative stress, are discussed as major drivers of cataract formation (Sweeney and Truscott, 1998; Moffat et al., 1999; Truscott, 2005; Abdelkader et al., 2015). The observable opacities may vary from almost non-visible punctual dots to a complete obfuscation of the lens, resulting in complete blindness (Thylefors et al., 2002).

#### Diagnosis and treatment

**Figure 1:**
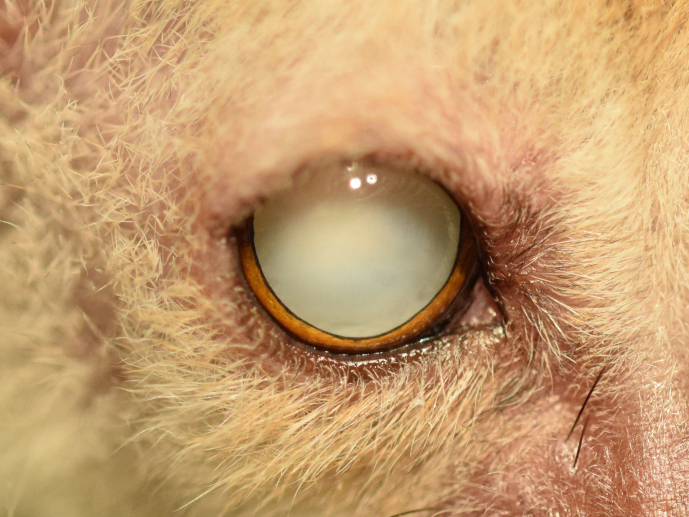
Twelve-year old animal with induced mydriasis. Both lenses are affected by mature cataracts.

For the diagnosis of cataracts a complete mydriatic stage is necessary, which can be reached by the application of mydriatic eye drops (Mydrum${}^{\text{®}}$, Chauvin ankerpharm GmbH, Berlin, Germany). A classification system is provided by the WHO (Thylefors et al., 2002), for which a slit lamp is needed to define the location and expansion of the cloudiness. In the final stages, as seen in Fig. 1, a slit lamp investigation may not be necessary, since the whole lens is affected (mature cataract). Nowadays only surgical treatments of cataracts are known to restore full functionality by using lens extraction (Wilkie and Colitz, 2009). This technique has been successfully applied in NHPs like gibbons (Duy et al., 2010). Although accommodation is subsequently only possible with artificial lenses, treated patients without implants are at least able to see a blurred picture of the environment.

#### Incidence

Cataracts represents the most frequent pathological finding in all mouse lemur colonies investigated so far. In our screenings, 29 out of 100 individuals between the age of 3.8 and 11.7 years showed beginning stages of cataracts (incipient cataract) located in the posterior and/or anterior lens cortex and/or under the lens epithelium. Using the classification system by Thylefors et al. (2002) these stages represent grade COR-0. Incipient cataracts was also the most frequent form (37 out of 218) described by Beltran et al. (2007).

**Figure 2:**
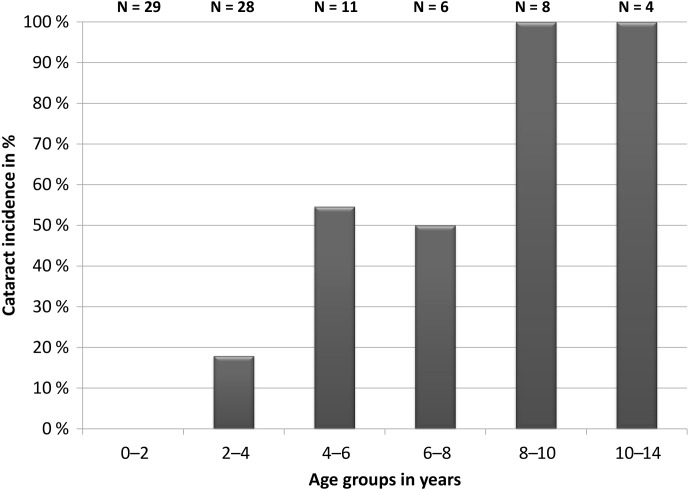
Incidence of cataracts in different age categories of mouse lemurs. N is the number of all investigated animals in this age group.

In our screening, bilateral mature cataracts (grade NUC-9 Thylefors et al., 2002) was diagnosed in five animals (out of 100 animals) ranging from 7.8 years to 13.6 years (see Fig. 1). Beltran et al. (2007) mentioned seven cases of mature cataracts (among 218 animals) in their investigation. In humans, this form of cataract is typically seen in old individuals and age is thought to be the main factor for its development (Moffat et al., 1999; Truscott, 2005; Michael and Bron, 2011). UV light is considered one of the most serious risk factors for cataract formation. We measured UV light by using a UV light meter in all rooms of our colony and found a value of 0 $W\;m^{-2}$ (Dubicanac et al., 2017). Thus, UV light can be excluded as a risk factor in our colony. However, UV light was not measured in Beltran et al. (2007). Diagnosed cortical cataract forms in our colony were not progressive during our study period., We presume that older mouse lemurs (approx. >8 years) most likely suffered from age-related nuclear cataracts due to the much higher life expectancy of mouse lemurs in captivity than in the wild (Zimmermann et al., 2016). A screening that was performed in 2014 showed that a 50 % cataract incidence was reached between 4 and 6 years, which increased to 100 % incidence at 8 years, indicating a high age dependency of cataracts (Dubicanac et al., 2017; see also Fig. 2). Among five old animals with mature cataracts (5 animals a, b, c, d and e can form a maximum of 10 dyads among each other, e.g. a–b, a–c, a–d, a–e, c–d, c–e, etc.), there were only two close kin relationships (r=0.5, full siblings) and two distant kin relationships (r=0.125, cousins). This distribution indicates a rather general appearance than a clear lineage dependency. However, a genetic impact on age-related nuclear cataracts has been described in humans (Hejtmancik and Kantorow, 2004) and should not be completely excluded for mouse lemurs.

#### 3.2.2 Nuclear sclerosis

#### Definition

Nuclear sclerosis (NS) is a physiological cloudiness of the lens (Glover and Constantinescu, 1997; Gelatt et al., 2012; Maggs et al., 2012) and must be distinguished from cataracts. Nuclear sclerosis represents a physiological process of the aging lens and is caused by an increased density of lens fibres in the lens core (Gwin and Gelatt, 1981; Glover and Constantinescu, 1997; Keil and Davidson, 2001; Gelatt et al., 2012; Maggs et al., 2012). It has an influence on the formation of presbyopia by restricting the lens-focusing ability (Glover and Constantinescu, 1997; Gelatt et al., 2012; Maggs et al., 2012). Polygenetic and environmental factors are discussed as possible reasons for NS formation in humans (Klein et al., 2005a).

#### Diagnosis and treatment

The diagnosis of nuclear sclerosis requires a complete mydriatic stage, which can be reached by the application of mydriatic eye drops (Mydrum${}^{\text{®}}$, Chauvin ankerpharm GmbH, Berlin, Germany). A slit lamp is needed to define the degree of expansion but without using other methods it may lead to a misdiagnosis of a nuclear cataract (Maggs et al., 2012). Furthermore, nuclear sclerosis in mouse lemurs appears particularly dense when investigated with a slit lamp (Dubicanac et al., 2017). A funduscopic investigation is necessary to distinguish NS from cataracts, e.g. by using an indirect ophthalmoscope (Omega 100; Heine, Ettenheim, Germany). In the case of NS, the cloudiness will not visually block details of the retina, while cataracts will not allow retinal structures to be seen when lying within the visual axis. Usually no medical treatment is conducted, since nuclear sclerosis alone has not been found to cause major damage to eye structures. Nevertheless, advanced stages with high density impair visual performance and the only known treatment is surgical lens extraction (Glover and Constantinescu, 1997; Wilkie and Colitz, 2009; Gelatt et al., 2012).

#### Incidence

**Figure 3:**
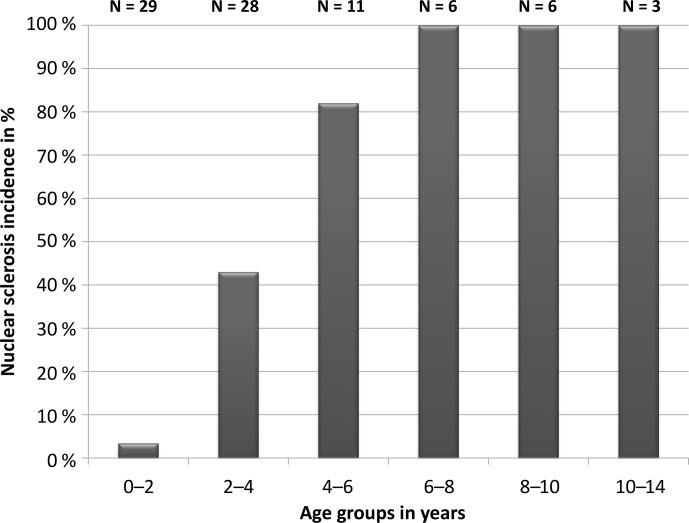
Incidence of nuclear sclerosis in different age categories of mouse lemurs. N is the number of all investigated animals per age group.

Nuclear sclerosis represented the most frequent ocular finding in our investigation, and the youngest animal diagnosed with NS was 1.8 years old (Table 1). Beltran et al. (2007) reported that NS does not represent the first visible cloudiness, without mentioning case numbers (Beltran et al., 2007). Frequent incidences of NS can also be seen in humans (Klein et al., 1992; Klein, 1993; Leske et al., 2002) and other mammals like dogs (Gelatt et al., 2012; Maggs et al., 2012). However, both studies were performed on colonies which originated from different ancestors in Madagascar. Therefore, a different genetic background could potentially represent a risk factor for NS formation, as it is already described in humans (Klein et al., 2005a). However, NS in mouse lemurs in our colony did not show aggregations in certain lineages, therefore making a genetic influence on NS formation unlikely. Animals younger than 2 years usually did not display signs of NS, whereas more than 40 % of individuals between 2 and 4 years developed NS (N=28), and all mouse lemurs older than 6 years showed certain stages of NS, as seen in Fig. 3. In conclusion, age seems to be the predominant driver of NS formation for mouse lemurs.

#### 3.2.3 Iris synechia/pupil seclusion

#### Definition

Iris synechia and pupil seclusion are diseases affecting the iris muscle. Iris synechia and the more severe form pupil seclusion (Seclusio pupillae) describe the adhesion of the iris muscle to neighbouring structures (Gelatt et al., 2012; Maggs et al., 2012). The reasons are usually inflammatory processes in the eye, often caused by traumatic interventions like surgery (Rowsey and Gaylor, 1980; Kim et al., 2009; Hilgert et al., 2012). The pupillary edge can be attached partially to the cornea (anterior synechia) or to the lens (posterior synechia) but also throughout the entire pupillary margin, resulting in pupil seclusion. While partial synechia impairs the pupillary reflex and accommodation ability, pupil seclusion makes a pupillary reflex impossible, impairs the ability of accommodation and raises the risk of increased intraocular pressure.

#### Diagnosis and treatment

For the diagnosis of iris synechia and/or pupil seclusion, the pupillary reflex has to be tested and a slit lamp investigation has to be performed. Since an absence of the pupillary reflex can also be caused by neurological dysfunctions, a slit lamp investigation is necessary to evaluate the pupils' edges in detail. In humans, the treatment may include cycloplegics (dilatation of the pupil), laser iridotomy (laser induced opening through the iris) or surgical synechiolysis (detaching the iris by using conventional surgical instruments), but prevention is still preferable, e.g. by using cycloplegic medication before synechiae can form (Sato, 1953; Sourdille, 1954; Jun Hu and Chen, 2010).

#### Incidence

**Figure 4:**
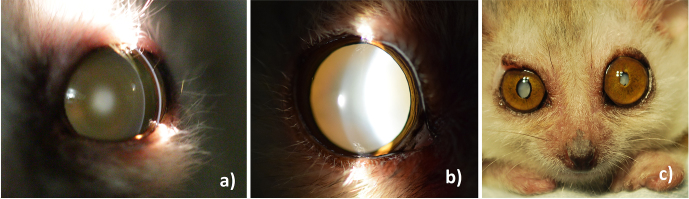
Typical development of age-related ocular changes in mouse lemurs. **(a)** A 3-year old animal with nuclear sclerosis, **(b)** an 8-year old animal with mature cataract, **(c)** a 12-year old animal with both lenses affect by mature cataracts. The right eye shows additional partial iris posterior synechia, the left eye shows pupil seclusion.

Five out of 100 animals were diagnosed with posterior pupil seclusion (n=5) from which one animal showed both iris posterior synechia and posterior pupil seclusion (n=1) in our screening (Table 1). The age at diagnosis was ≥10 years and all individuals were additionally affected by mature cataracts as seen in Fig. 4. Since no young mouse lemur was affected, age can be suggested as the most important factor for the formation of posterior iris synechia and/or pupil seclusion. Inflammatory processes can be excluded for four of the five animals. However, all animals with iris synechia/pupil seclusion were also affected by mature cataracts. Thus, our findings support Beltran et al. (2007), who observed 15 cases of iris synechia and pupil seclusion associated with cataracts.

#### 3.2.4 Hyphema

#### Definition

Hyphema is a clinical sign. It is defined as the accumulation of blood cells in the anterior eye chamber usually caused by damage to the uveal tract or to the globe (Gelatt et al., 2012; Maggs et al., 2012). The most common reasons for its appearance is traumatic injury (Swan, 1973; Speakman, 1975; Sandhu et al., 2016). Nevertheless, a high diversity of ocular diseases, e.g. tumours, genetic predispositions and inflammatory processes, can also cause increased permeability of blood vessels, allowing blood cells to enter the intraocular fluid (Arentsen and Green, 1975; Brenkman et al., 1977; Mcdonald et al., 1989; Akpek and Gottsch, 2000; Mitchell, 2006). Additionally, substances that modify platelet or thrombin function, e.g. aspirin and warfarin, can cause hyphema (Koehler and Sholiton, 1983; Schiff, 1985). Complications are uncommon but can result in an increase of intraocular pressure, iris synechia and the atrophy of the optic nerve (Coles, 1968; Rakusin, 1972; Read and Goldberg, 1974; Maggs et al., 2012).

#### Diagnosis and treatment

**Figure 5:**
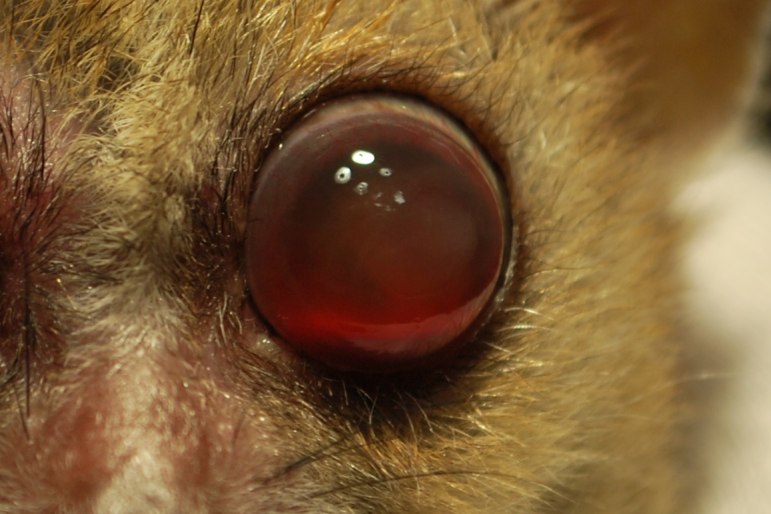
Eight-year old individual with bilateral blood accumulation (Hyphema) in the anterior eye chamber, presumably as a result of inflammatory processes of the uvea (uveitis). The blood cells accumulate at the bottom of the anterior eye chamber in order to follow gravity.

A slit lamp serves best, since structures within the anterior eye chamber can be seen in detail. Usually the blood cells follow gravity and accumulate at the bottom of the eye chamber as seen in Fig. 5. The treatment in veterinary medicine depends on and aims at its primary cause (Telle and Betbeze, 2015). To prevent more blood entering the eye chamber, aminocaproic acid or tranexamic acid can be used, but this also slows down the clearing process of the existing hyphema (Pandolfi et al., 1966). Steroid medication can be useful for treating inflammatory processes, if they are identified as the causing factor of hyphema, and to prevent secondary hemorrhage (Ohrstrom, 1972; Yasuna, 1974).

#### Incidence

Incidence for this ocular finding in mouse lemur colonies is rare (Table 1). During our investigations we identified one case of hyphema (also one case in Beltran et al., 2007) which was accompanied by inflammatory processes of the sclera (scleritis), cornea oedema and corneal vascularisation. In both Beltran's study and ours, hyphema was cataract associated. The animal in our study was 8 years old and both eyes were affected. Due to its rarity, the role of genetic background and of age for this disorder remains unclear. However, it is known for dogs that congenital defects can cause hyphema, e.g. persistent hyaloid artery, Collie eye anomaly and vitreoretinal dysplasia (Mitchell, 2006; Gelatt et al., 2012).

#### 3.2.5 Intraocular hypertension

#### Definition

Intraocular hypertension is a clinical sign. It describes the increased intraocular pressure of the eye, caused by accumulation of aqueous humour (Gelatt et al., 2012; Maggs et al., 2012). Aqueous humour is constantly produced in the ciliary body and leaves the eye through the trabecular meshwork located in the iridocorneal angle. Inflammatory processes, blood pressure and tumours can directly influence intraocular pressure (IOP) or damage involved structures and lead to drastic elevations of IOP (Kinshuck, 1991; Shields et al., 2001; Klein et al., 2005b; Radcliffe and Finger, 2009). Long exposure to increased IOP is one of the main risk factors for glaucoma development (Kass et al., 2002).

#### Diagnosis and treatment

Tonometry is the only known practical way to determine IOP in non-anaesthetised animals. Many different methods of tonometry exist today, and it has been shown that for mouse lemurs the TonoVet${}^{\text{®}}$ provides reliable measurements (Dubicanac et al., 2016). Physiological IOP in mouse lemurs ranges (as in other species) around 20.3±2.8 mmHg (Dubicanac et al., 2016). Constantly higher IOP needs medical intervention to prevent secondary disorders like glaucoma (Kass et al., 2002). In postoperative cases or cases of inflammatory processes like uveitis, treatment of increased IOP in humans and small animals can be the topical application of carbonic anhydrase inhibitors (Brinzolamide) and/or timolol (Kaback et al., 2008; Gelatt et al., 2012; Ornek et al., 2013; Takeuchi et al., 2017).

#### Incidence

**Figure 6:**
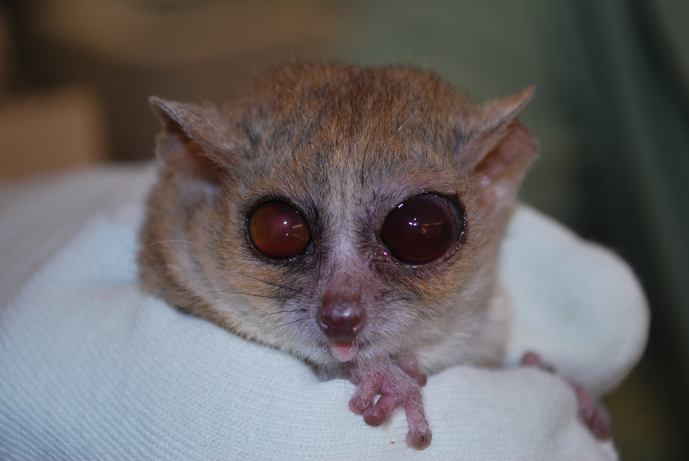
Eight-year old individual (same individual as in Fig. 5) with an enlarged left eye (exophthalmos).

This ocular finding is rare in mouse lemur colonies (Table 1). During our study, pathologically increased IOP (> 30 mmHg) was found in only 1 out of 100 studied animals. Both eyes of one 8-year old animal showed an IOP of >40 mmHg, which was caused by a preceded inflammation and dense hyphema (see Fig. 6). In the study of Beltran et al. (2007), IOP values have not been measured due to difficulties in the use of applanation tonometry in non-anaesthetised animals. Due to its rarity, the influence of genetic background and of age cannot not be determined. In humans, genetic background (Van Koolwijk et al., 2007; Van Koolwijk et al., 2012; Gao et al., 2016) as well as age are suggested to affect IOP with a general increasing tendency with increasing age, although it seems to depend on the investigated population (Colton and Ederer, 1980; Shiose, 1990; Dielemans et al., 1995; Bonomi et al., 1998). Studies performed on healthy, aging mouse lemurs did not support a general increase of IOP with age (Dubicanac et al., 2016).

#### 3.2.6 Exophthalmos

#### Definition

Exophthalmos is a clinical sign. It is described as the abnormal enlargement and bulging of the eye anteriorly out of orbit (Gelatt et al., 2012; Maggs et al., 2012). Underlying causes are usually inflammations (Bowers et al., 2009; Morax and Badelon, 2009; Hafidi and Daoudi, 2013), endocrinopathies (Werner, 1969; Morax and Badelon, 2009; Giugni et al., 2013) or neoplastic processes (Chi et al., 2009; Hafidi and Daoudi, 2013; Bouzidi et al., 2015; Vardarli et al., 2016) as well as heredity causes (e.g. brachiocephalic dog breeds) and trauma (Chousterman et al., 2014; Manousaridis et al., 2016).

#### Diagnosis and treatment

In severe cases exophthalmos can be seen by optical inspection alone. An exophthalmometer can be used to define more inconspicuous cases. The treatment depends on the underlying cause. Inflammatory processes can be treated with a combination of antibiotics and topical steroid medication, whereas neoplastic causes usually need surgical treatment, as performed on small animals, for example (Gelatt et al., 2012). If increased IOP is involved, topical medication can be used, such as carbonic anhydrase inhibitors (Brinzolamide) and/or timolol (Kaback et al., 2008; Gelatt et al., 2012; Ornek et al., 2013; Takeuchi et al., 2017).

#### Incidence

This ocular finding is rare in mouse lemur colonies (Table 1). During our study, we identified 1 animal (8 years old) out of 100 animals as being affected. Its exophthalmos can be seen in Fig. 6. The enlargement followed an inflammation of the eye bulb accompanied by extremely high IOP. Due to its rarity, the influence of genetic background and age could not be determined. Nevertheless, in humans a special form of exophthalmos (buphthalmia), which is caused by an increased IOP in the prenatal or early postnatal life period, is described to have a genetic background (Faiq et al., 2013), but it is rarely seen in adults (Alves et al., 2012). However, one case of this special form, which also was described to be cataract associated, was diagnosed by Beltran et al. (2007) in mouse lemurs.

#### 3.2.7 Phthisis bulbi

#### Definition

Phthisis bulbi is the final stage of severe diseases of the globe. It describes a shrunken and non-functional eye (Gelatt et al., 2012; Maggs et al., 2012). Due to scar tissue and severe damage of the ciliary body, the production of aqueous humour is interrupted and consequently important eye structures atrophy. Phthisis bulbi can occur, for example, after severe inflammation (Atipo-Tsiba and Kombo Bayonne, 2016) or injury (Menezo and Illueca, 1983; Soares and França, 2010).

#### Diagnosis and treatment

The shrunken eye can be identified by simple visual examination; good light conditions (e.g. slit lamp) are necessary. Phthisis bulbi represents the final stage after severe complications and therefore no medical treatment for functional restoration is known today.

#### Incidence

This ocular finding is rare in mouse lemur colonies (Table 1). We identified 1 out of 100 studied animals with bilateral phthisis bulbi. The age of this animal was 8 years and phthisis bulbi occurred bilaterally as a consequence of previous severe inflammation of both eyes. Beltran et al. (2007) also described one case of phthisis bulbi. Due to its rarity in mouse lemurs and other species, genetic disposition and age dependencies are not known yet.

#### 3.2.8 Ectopia lentis

#### Definition

Ectopia lentis is a disease of the lens and/or its surrounding structures. It is described as the displacement of the lens from its normal position (lens luxation) (Gelatt et al., 2012; Maggs et al., 2012). The lens is surrounded by the ciliary body and connected to it by suspensory ligaments (zonula fibres). In cases of secondary lens luxation (e.g. after severe traumatic injuries, cataracts, neoplasia, inflammation of the ciliary body or in cases of primary lens luxation, such as congenital-dependent fragile suspensory ligaments as in dogs; Davidson and Sr, 1999), the zonula fibres may detach from the lens capsule. This may happen partially or completely and may shift the lens towards the cornea (anterior lens luxation) or towards the vitreous body (posterior lens luxation).

#### Diagnosis and treatment

For diagnosis, a slit lamp, as well as mydriatic eye drops, is necessary to reach a complete mydriatic stage. If the lens is partially dislocated, the margin of the lens capsule from the detached side usually can be seen to be pushed towards the centre of the pupil. In the case of complete anterior lens luxation, usually the lens is visible in the anterior eye chamber without inducing a mydriatic stage. To diagnose a posterior lens luxation a complete mydriatic stage is necessary with the lens usually lying on the bottom of the eye (Davidson and Sr, 1999). The only known treatment of secondary complications caused by lens luxation is lens extraction and topical medication to prevent inflammation or intraocular hypertension and consequently glaucoma (Davidson and Sr, 1999).

#### Incidence

**Figure 7:**
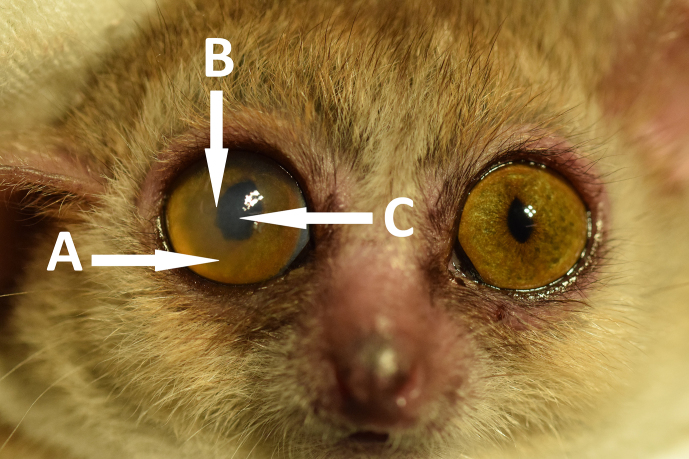
Three-year old mouse lemur with a unilateral complete anterior lens luxation on the right side. Because of the complete transparency of the lens, the luxation is only indicated by an incomplete miosis as well as the blurred iris (A) and distorted iris margin (B). The right cornea shows additional edema (C).

This ocular finding is rare in mouse lemur colonies (Table 1). In our study one 3-year old animal out of 100 animals was diagnosed with a complete anterior lens luxation; see Fig. 7. No previous or following inflammation as well as no cataract was visible. Cornea oedema manifested after 1 week of diagnosis. Although we presume traumatic causes, genetic predisposition was shown for terrier breeds (Davidson and Sr, 1999) and cannot be totally excluded. Beltran et al. (2007) diagnosed one case of posterior lens luxation which was also cataract associated. Since only two cases are described in mouse lemur colonies (Table 1), however, genetic predispositions and age dependencies cannot be clarified.

#### 3.2.9 Corectopia

#### Definition

Corectopia is a disease of the iris muscle. It describes the eccentric displacement of the pupil (Gelatt et al., 2012; Maggs et al., 2012). It is usually caused by congenital reasons (Ennis et al., 2006) and associated with other pathological conditions like myopia and ectopia lentis (Milea and Burillon, 1999) and chromosomal as well as central nervous system abnormalities (Ennis et al., 2006). Nevertheless, causes are also quite frequently idiopathic (Kumar et al., 2000; Brockmann et al., 2016).

#### Diagnosis and treatment

To be able to investigate the position of the pupil, a slit lamp or any other good light source can be used which provides a clear picture of the iris. The pupil should be in miosis since a deviated pupil could falsify the position of the pupil. Usually the treatment of associated abnormalities is more important than the correction of corectopia itself. Nevertheless, surgical treatments as well as those using a Nd:YAG laser have been applied in humans (Griener and Lambert, 1999; Brockmann et al., 2016).

#### Incidence

This ocular finding is rare in mouse lemur colonies (Table 1). As a result the genetic predisposition and age dependency cannot be determined yet. Nevertheless, genetic predisposition is likely, since this is the most frequently described cause described for humans (Ennis et al., 2006).

#### 3.2.10 Trichiasis

#### Definition

Trichiasis is a disease of the eyelashes. It is described as the misdirected position of eyelashes, which point towards the eye and irritate the cornea's surface (Gelatt et al., 2012; Maggs et al., 2012). These irritations may have almost no impact on the visual performance but can lead to complete blindness. The leading cause for trichiasis in humans is an infection called trachoma which is caused by *Chlamydia trachomatis*. It is one of the most frequent causes of infectious blindness in humans (Burton et al., 2015). Severe inflammatory processes may cause scarring of the inner surface of the eyelids and cause deformations of the eyelid structure called entropion, which leads to trichiasis. Other causes of human trichiasis can be congenital, autoimmune, traumatic and secondary after enophthalmos (Kirkwood and Kirkwood, 2011). Entropion, and consequently trichiasis, is also known to be present in animals like dogs and cats (Williams and Kim, 2009).

#### Diagnosis and treatment

A slit lamp is necessary to examine the exact position of the eyelashes. Minor trichiasis comprises cases in which less than five eyelashes are misdirected and major trichiasis comprises cases in which five or more are pointing towards the cornea (Ferreira et al., 2010). The treatment includes the surgical correction of misdirected eyelashes or the affected eyelid if entropion is additionally present. Techniques which can be used are epilation, electrolysis, laser photoablation, cryotherapy and common surgery (Kirkwood and Kirkwood, 2011).

#### Incidence

**Figure 8:**
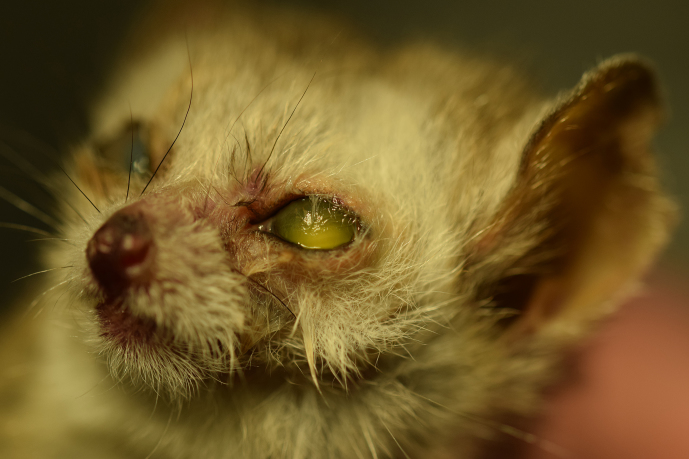
Post-mortem picture of a 10-year old animal affected by unilateral trichiasis of the upper eyelid on the left eye.

This ocular finding is rare in mouse lemur colonies (Table 1). One case of trichiasis has been observed in a 10-year old animal in our study of 100 animals, which can be seen in Fig. 8. Beltran et al. (2007) did not report any case of trichiasis. Thus, it was not possible to examine whether trichiasis is based on congenital factors or develops with increasing age.

#### 3.2.11 Cornea dystrophy

#### Definition

Cornea dystrophy represents a clinical subsection of corneal diseases. Cornea dystrophy is multifactorial and includes a high variety of clinical manifestations, while impairments can vary from minimal impact to complete blindness. Cornea dystrophy is defined as an inherited disorder of the cornea itself, which forms spontaneous bilateral opacities and usually has no systemic manifestation (Weiss et al., 2008; Klintworth, 2009; Weiss et al., 2015).

#### Diagnosis and treatment

A slit lamp is necessary for basic evaluation. For a first visual estimation of the exact form of dystrophy, literature on small animals and humans can be used (Klintworth, 2009; Gelatt et al., 2012). More precise definitions can only be made by using surgical excised corneal tissue and molecular genetic analysis (Klintworth, 2009). Since dystrophies are inherited, corneal defects usually represent symptoms which can only be treated symptomatically. Some dystrophies have very low impact on vision and therefore do not need medical intervention. If treatment is necessary, it depends on its symptoms and may range from topical medical application (e.g. antibiotics, cycloplegics) to surgical intervention (e.g. deep lamellar endothelial keratoplasty, phototherapeutic keratectomy) and have variable clinical success at least in humans (Klintworth, 2009).

#### Incidence

This ocular finding is rare in mouse lemur colonies (Table 1). Beltran et al. (2007) mentioned three cases of corneal dystrophy without providing details on genetic background or age of the mouse lemurs. We could not identify corneal dystrophies during our own investigations. Thus, it is not possible to assess the effect of genetic background or age on this eye pathology. Since corneal dystrophies are under close investigation in humans, the genetic background in several forms is already well defined (Weiss et al., 2008; Klintworth, 2009). This makes the genetic background for corneal dystrophies in mouse lemurs likely and the investigation of concerned lineages interesting for future studies.

#### 3.2.12 Cornea degeneration

#### Definition

Corneal degeneration is a disease of the cornea. It is a gradual deterioration of previously functional tissue (Gelatt et al., 2012; Maggs et al., 2012). In contrast to corneal dystrophies, the causes of cornea degeneration are not congenital and can affect only one eye (Roszkowska and Wylegala, 2015). Environmental influences like UV light, oxidative stress, systemic disorders and age-related processes are discussed as possible causes in humans (Friedlaender and Smolin, 1979; Sacca et al., 2013). In cases of age-related processes, corneal degenerations may occur bilaterally but asymmetrically (Roszkowska and Wylegala, 2015).

#### Diagnosis and treatment

A slit lamp is necessary for the exact determination and localisation of corneal changes. Usually the visible changes in the cornea are opacities, cornea deformation or tissue thinning. A visual summary of the different forms of degenerations in small animals and humans can be seen in Gelatt, Gilger and Kern (2012) or Roszkowska and Wylegala (2015). While age-related corneal degenerations usually do not need medical therapy, other forms often represent symptoms which require treatment of the underlying systemic or local disorder (Roszkowska and Wylegala, 2015). Symptomatic treatment includes contact lenses, eyelid hygiene, lubricants and anti-inflammatory eye drops if lacrimation is disturbed. Severe cases may need surgical intervention, such as corneal grafting, at least in humans (Roszkowska and Wylegala, 2015).

#### Incidence

Nine cases of corneal degeneration were described by Beltran et al. (2007), of which five were associated with cataracts, but no cases were found in our study. The age and family relationship of the animals is not known and therefore nothing is known about the genetic background. However, heredity factors are not likely to initiate corneal degeneration, while age-related processes may cause it (e.g. keratoconus) and should be considered seriously, as described in humans (Friedlaender and Smolin, 1979; Roszkowska and Wylegala, 2015).

#### 3.2.13 Chorioretinitis

#### Definition

Chorioretinitis is a clinical sign. It follows the inflammation of two eye structures called the choroid and retina (Gelatt et al., 2012; Maggs et al., 2012). The causes of chorioretinitis in humans are usually bacterial (Yang et al., 2015; Molina-Socola et al., 2016), viral (Anninger et al., 2003; Eidsness et al., 2005) as well as most frequently parasitic infections with the intracellular parasite *Toxoplasma gondii*, which can be transmitted horizontally as well as vertically (congenital) (Gump and Holden, 1979; Montoya and Remington, 1996; Datta and Banerjee, 2003; Hall et al., 2009). Nevertheless, cases linked to HIV are also known (Garcher et al., 1990).

#### Diagnosis and treatment

For the diagnosis of chorioretinitis a complete mydriatic stage is necessary, which can be reached by the application of mydriatic eye drops (Mydrum${}^{\text{®}}$, Chauvin ankerpharm GmbH, Berlin, Germany). An ophthalmoscopic investigation has to be performed to investigate the retina. Summaries on possible fundus findings in small animals and humans are available here (Gelatt et al., 2012; Maggs et al., 2012; Yang et al., 2015). The treatment depends on the primary cause. Usually a combination of antibiotics and corticosteroids is necessary, and pyrimethamine can be added if toxoplasmosis is present (Psilas et al., 1990).

#### Incidence

This ocular finding is rare in mouse lemur colonies (Table 1). Beltran et al. (2007) described one inactive case of chorioretinitis (scars on the retina), which showed cataract association. In our colony no active and no inactive chorioretinitis could be found. Thus, it was not possible to examine whether chorioretinitis is based on congenital factors or develops with increasing age.

## 4 Conclusions

This review shows that mouse lemurs show various ocular impairments which may distort vision. Impairments of vision often lead to changes or disturbances in the circadian activity rhythm that can be recognized by irregular feeding bouts or locomotor activities during the periods of daylight (Zimmermann, Radespiel, Dubicanac, personal observation). A genetic or age-related dependency could not be verified for most ocular findings due to a low number of cases., Across mouse lemur colonies, the most widespread and obvious ocular findings were NS and cataracts. Both increase with increasing age (see Sects. 3.2.1/3.2.2). Ophthalmological investigations should therefore be performed routinely on animals older than 5 years and especially prior to their use for studies which require normal vision. Iris posterior synechia has been described from different colonies and seems highly age dependent and cataract associated (see Sect. 3.2.3). Although other ocular findings (e.g. lens luxation, trichiasis, hyphema and buphthalmia) do not occur as frequently as the previously mentioned ones, they may still require special ophthalmological examinations and treatments to ensure animal well-being.

## Supplement

10.5194/pb-4-215-2017-supplement The supplement related to this article is available online at: https://doi.org/10.5194/pb-4-215-2017-supplement.

## Data Availability

Data used in this review are based partially on data which are published and freely accessible in Dubicanac et al. (2016, 2017). All additional data are visible in Table 1. Additionally we provide a data set in an Excel file as a Supplement to complete the missing information.
