# Supplementary material for: A review on ocular findings in mouse lemurs: potential links to age and genetic background
Source: Primate Biol. 2017 Oct 27;4(2):215–28. doi: 10.5194/pb-4-215-2017 (PMC7041539; doi:10.5194/pb-4-215-2017)
Supplement: The supplement related to this article is available online at: https://doi.org/10.5194/pb-4-215-2017-supplement. [file pb-4-215-supplement.zip › pb-4-215-2017-supplement-title-page.pdf]

Supplement of Primate Biol., 4, 215–228, 2017
https://doi.org/10.5194/pb-4-215-2017-supplement
© Author(s) 2017. This work is distributed under
the Creative Commons Attribution 3.0 License.

Supplement of

A review on ocular findings in mouse lemurs: potential links to age and genetic background

Marko Dubicanac et al.

Correspondence to: Elke Zimmermann (elke.zimmermann@tiho-hannover.de)

- pb-4-215-2017-supplement-title-page.pdf
- DataSet_PB.xlsx

The copyright of individual parts of the supplement might differ from the CC BY 3.0 License.
